# Evidence for the Role of a Second Fc-Binding Receptor in Placental IgG Transfer in Nonhuman Primates

**DOI:** 10.1128/mbio.00341-23

**Published:** 2023-03-22

**Authors:** Yvonne J. Rosenberg, Tracy Ordonez, Urjeet S. Khanwalkar, Philip Barnette, Shilpi Pandey, Iara M. Backes, Claire E. Otero, Benjamin S. Goldberg, Andrew R. Crowley, David A. Leib, Mariya B. Shapiro, Xiaoming Jiang, Lori A. Urban, Jonathan Lees, Ann J. Hessell, Sallie Permar, Nancy L. Haigwood, Margaret E. Ackerman

**Affiliations:** a PlantVax, Inc., Rockville, Maryland, USA; b Oregon National Primate Research Center, Oregon Health & Science University, Beaverton, Oregon, USA; c Thayer School of Engineering, Dartmouth College, Hanover, New Hampshire, USA; d Geisel School of Medicine, Dartmouth College, Hanover, New Hampshire, USA; e Department of Pediatrics, Weill Cornell Medicine, New York, New York, USA; University of North Carolina at Chapel Hill

**Keywords:** plant and mammalian antibodies, primates, mice, placental transfer, FcRn, FcγR, Fcγ receptors, human immunodeficiency virus, human monoclonal antibodies, mouse models, nonhuman primate models, placental immunology, plant production of antibodies

## Abstract

Transplacental transfer of maternal antibodies provides the fetus and newborn with passive protection against infectious diseases. While the role of the highly conserved neonatal Fc receptor (FcRn) in transfer of IgG in mammals is undisputed, recent reports have suggested that a second receptor may contribute to transport in humans. We report poor transfer efficiency of plant-expressed recombinant HIV-specific antibodies, including engineered variants with high FcRn affinity, following subcutaneous infusion into rhesus macaques close to parturition. Unexpectedly, unlike those derived from mammalian tissue culture, plant-derived antibodies were essentially unable to cross macaque placentas. This defect was associated with poor Fcγ receptor binding and altered Fc glycans and was not recapitulated in mice. These results suggest that maternal-fetal transfer of IgG across the three-layer primate placenta may require a second receptor and suggest a means of providing maternal antibody treatments during pregnancy while avoiding fetal harm.

## INTRODUCTION

Transcytosis of maternal IgG across the placenta, which occurs predominantly during the latter half of pregnancy, provides the fetus and newborn with passive protection against infectious diseases (1, 2). Though their anatomies vary widely, placentas have evolved in all mammals except egg-laying monotremes to support the developing offspring in terms of nutrients, oxygen import, waste disposal, and immune protection (3). Morphologically diverse mammalian placentas can be classified according to the number and type of cell layers that separate the bloodstreams of the mother and conceptus (3), which are in turn associated with differences in maternal IgG delivery to neonates. Prenatal placental IgG transfer in primates is essentially the sole route of maternal IgG transfer to fetal circulation, while the dominant mode of IgG transfer in rodents is through postnatal uptake of IgG from breast milk via the intestine. Additionally, while murine models are frequently used in studies of antibody (Ab) transfer, the mouse chorioallantoic placenta does not play a role in IgG transfer (4), which is instead mediated by the mouse yolk sac (5–7). In contrast, the human placenta has evolved as an organ consisting of three anatomical barriers (8): (i) the outermost syncytiotrophoblast cell barrier, (ii) the villous stroma containing placental fibroblasts and Hofbauer cells, and (iii) fetal endothelial cells. Despite its greater complexity, the human placenta provides such efficient delivery of IgG that, provided there are durable levels in maternal serum over the second and third trimesters, levels of both natural and recombinant IgG in neonates typically exceed those observed in their mothers (9).

Based on a diversity of experimental approaches, mammals have been shown to utilize the highly conserved FcRn heterodimer (9–16), a member of the major histocompatibility complex class I (MHC-I) family of proteins found on the syncytiotrophoblast layer in humans, as the primary and potentially the sole receptor involved in maternal-fetal transfer of IgG. However, recent studies (17, 18) have suggested that during the evolution of the three-layer primate placenta, the transfer mechanism may have diverged to involve another receptor that contributes to transfer across the final placental layers (19, 20). Based on expression, candidate IgG receptors include FcγRIIIa, which is expressed on syncytiotrophoblasts, FcγRIIb on fetal endothelial cells (19–21), and FcγRI, FcγRIIb, and FcγRIIIa on placental fibroblasts and Hofbauer cells (22, 23). Intriguingly, studies comparing maternal and neonatal IgG pools have long shown variability in transfer efficiency by IgG subclass that appears to be poorly explained by FcRn affinity (24). Additionally, some recent studies have suggested that posttranslational modifications, including the IgG Fc glycoforms identified, which is known to impact FcγR binding, may also play a role in placental transfer efficacy of human IgG (8, 17, 18). While other studies have reported similar trends in glycoprofile differences between maternal and cord blood IgG (9, 10, 25), they have at times drawn opposing conclusions, in part based on mechanistic tests performed in murine models. These discrepant conclusions highlight the potential value of studies to evaluate factors such as the impact of distinct IgG glycoforms in order to further elucidate mechanisms of antibody transfer in primates.

In this context, plant-derived proteins are essentially indistinguishable from those in animals and humans with respect to protein synthesis, secretion, and chaperone-assisted protein folding, together with the processes of posttranslational modification, such as signal peptide cleavage, disulfide bond formation, and the early stages of N-linked glycosylation. However, plant glycans are neither sialylated nor galactosylated and can have nonhuman complex glycans appended during the passage through the Golgi system, such as β1,2-xylose and core α1,3-fucose, which differ from mammalian core α1,6-fucose. Such variably glycosylated antibodies offer the opportunity to further investigate mechanisms of transgenerational antibody transfer in mammals.

To this end, despite the significant anatomical and immunologic differences between mice and primates, comparative analysis of placental IgG transfer between species has been limited, and observations from the mouse model have generally been presumed to extend to humans. Here, HIV envelope glycoprotein-specific broadly neutralizing human IgG1 antibodies (bNAbs) PGT121 (26) and VRC07-523 (27), including variants with Fc domain mutations designed to improve FcRn affinity (28, 29), were produced using the Nicotiana benthamiana p19 plant expression system. The placental transfer efficiency of these and mammalian cell-expressed counterparts, along with a modestly neutralizing antibody, 830A (30), were then compared in rhesus macaques and mice to assess whether IgG glycan profiles and receptor interactions beyond FcRn may play a prominent role in placental IgG transport. Surprisingly, results demonstrate that while plant- and mammal-produced antibodies exhibit comparable transfer efficiencies in mice, they are differentially transferred across macaque placentas, consistent with a two-receptor IgG transport model in primates. Importantly, the strong binding of plant bNAbs to FcRn, permitting desirable pharmacokinetics in humans while lacking the ability to efficiently cross the placenta, suggests that therapeutic plant-based MAb treatments against autoimmune diseases and cancer could be provided to the mother while avoiding transfer and preventing harm to the fetus.

## RESULTS

### Experimental design for primate studies.

Primate antibody studies utilized a macaque model involving subcutaneous (s.c.) passive administration of *N. benthamiana* plant-derived (denoted by the prefix “p” in the antibody name) or mammal-derived (denoted by the prefix “m” in the antibody name) human HIV envelope glycoprotein-directed bNAbs, and one monoclonal antibody (Mab) with only modest neutralizing activity. These antibodies had been previously shown to modulate or to prevent SHIV infection in macaques (29, 31–33). The bNAbs had either unmodified or engineered Fc domains with either YTE (34) or LS (35) amino acid point mutations, which exhibit enhanced binding to FcRn at low but not neutral pH. These variants exhibit improved serum persistence and mucosal biodistribution in both humans and nonhuman primates (36). Six pregnant rhesus macaque dams close to parturition, as determined by sonogram, were used to assess the extent of antibody transfer via the placenta, with an initial goal of evaluating peripartum protection of the infants from infection. After vaginal birth, four of the six infants remained with their dams for the entire sampling period, receiving colostrum and breast milk, while two infants were moved to the nursery on the day of birth. Dams received, either singly or as cocktails, 5 mg/kg of body weight each of either plant-derived bNAbs pPGT121, pPGT121-YTE, and pVRC07-523-LS or mammal-derived CHO bNAb mPGT121 or HEK-293-derived bNAb m830A as controls, providing the ability to compare transfer of plant- and mammal-derived bNAbs in the same animal (Table 1). For some dams, delivery did not proceed on the anticipated timeline and a second subcutaneous (s.c.) bNAb injection was given to maintain high circulating levels of the bNAbs in the dams at parturition. Blood samples were collected from the pregnant dams for 14 to 28 days after injection and from the newborns for 72 h after birth. Levels of maternal and neonatal antibodies were assessed by quantitative enzyme-linked immunosorbent assay (ELISA) for comparison of the circulating levels of antibody in both dam and infant dyads over time.

**TABLE 1:** Summary of rhesus dam/infant placental transfer experiments^*a*^

Dyad	Dam	Infant	bNAb infusion^*b*^	Day(s) of infusion^*c*^	bNAb level at birth (μg/mL)	
					Dam	Infant
1	27435	36611^*d*^	pPGT121	−4	5.25	0.003
2	30060	36648^*d*^	pPGT121	−5, −1	18.53	0.011
3	27455	36176	pPGT121-YTE	−15, −1	19.6	0.1
4	25961	36310	pPGT121-YTE	−9, −5	28.5	0.001
5	31561	38294^*d*^	mPGT121	−6	13.00	0.71
			pVRC07-523-LS	−6	3.79	0.01
6	31306	38579^*d*^	pVRC07-523-LS	−6	3.95	0.02
			mPGT121	−6	12.77	1.47
			m830A	−6	19.17	3.5

a Placental transfer of human bNAbs produced in plant and mammalian cells across the placenta of 6 pregnant Macaca mulatta dams following one or two infusions of bNAbs prior to birth. The data shown summarize the comparison for matched time points in the dam and infant.

b In the bNAb names, the prefix “p” represents plant derived (Nicotiana benthamiana), and the prefix “m” represents mammal derived (CHO or HEK293 cells).

c Day(s) of infusion of 5 mg/kg of body weight of each bNAb relative to day of birth.

d Newborns remained with their respective dams and suckled colostrum and milk for the duration of the sampling period; those without symbols were moved to the nursery on the day of birth.

### Plant-derived antibodies exhibit poor placental transfer efficiency in primates.

**FIG 1:**
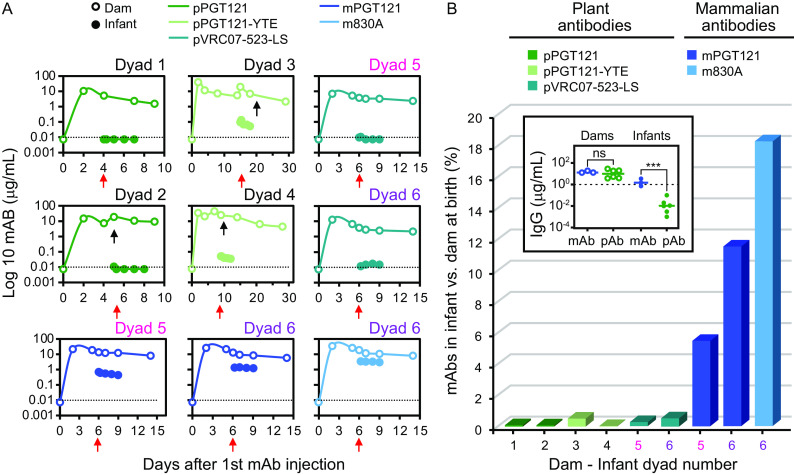
Plant-derived antibodies exhibit poor placental transfer efficiency in primates. Six dams were injected s.c. with one, two, or three plant (green)- and mammal (blue)-derived bNAbs prior to birth (dam-infant dyad numbers shown in Table 1). (A) Pharmacokinetic profiles of each Ab (in micrograms per milliliter) in the six dams (open circles) and infants (solid circles) following injections of the dams. Black arrows indicate second Ab infusions, and the red arrow indicates the infant’s day of birth. The dotted horizontal line indicates a concentration of 0.01 μg/mL. In dyads 5 (pink) and 6 (purple), dams were coinfused with two or three Abs, respectively. (B) Level of Ab in infants as a percentage of that observed in dams at the time of birth. The dyad number is shown on the *x* axis, and the Ab is indicated in color. *N. benthamiana*-derived Abs (shades of green [dyads 1 to 6]) include PGT121 and FcRn-engineered PGT121-YTE and VRC07-523-LS, while mammal-derived Abs include CHO-derived PGT121 and HEK-293-derived 830a (blue [dyads 5 and 6]). (Inset) Levels (micrograms per milliliter) of plant (p)- and mammal (m)-produced Abs across all Abs tested in dams (left) and infants (right) on the day of birth. Statistical significance was defined by unpaired two-tailed *t* test with Welch’s correction (***, *P* = 0.0004). Bars indicate medians. The dotted line indicates a concentration of 1 μg/mL.

In initial experiments (dyads 1 and 2), pPGT121 was infused singly into each of two pregnant dams, but unexpectedly resulted in very low levels of Abs in infants (Fig. 1A). This low transfer efficiency was not rescued by inclusion of Fc mutations that improve FcRn affinity, as administration of pPGT121-YTE to two additional dams resulted in similarly poor transfer to their infants. Across all four initial dyads, plant-derived antibody persisted, as expected in dams (Fig. 1A), but placental transfer was poor and reached only very low levels (0.003 to 0.1 μg/mL) in each of the four infants (Table 1).

In order to confirm these results while limiting the number of pregnant macaques required and to further address interanimal variability and differences in timing of delivery, two additional pregnant dams were coinfused with cocktails of both plant and mammalian bNAbs. The first was simultaneously administered both pVRC07-523-LS and mPGT121, and the other was treated with pVRC07-523-LS, mPGT121, and m830A. Consistent with results of monotherapy, the plant-expressed Abs were not efficiently transferred across the placenta in these dyads either; plant-derived antibodies were present at considerably lower levels (0.01 to 0.02 μg/mL) in infants than coadministered mammal-derived antibodies (0.71 to 3.5 μg/mL) (Fig. 1A).

Across all dyads, plasma antibody levels in infants as a proportion of levels observed in mothers at birth indicate a stark contrast between plant- and mammal-derived Abs (Fig. 1B). While mammalian Abs were well transferred across the placenta (5.46% to 18.26%), plant-derived Abs, even with YTE and LS mutations to increase affinity for FcRn, were poorly transferred (0.003 to 0.51%), representing a median 100-fold reduction in transfer efficiency of the plant relative to mammalian IgG forms.

In sum, we found that plant-derived pPGT121 was poorly transferred across the placenta in macaque dams compared to mammal-derived mPGT121, half-life-extending mutations did not restore efficient transfer, and this defect was generalizable to a second antibody with a distinct variable domain. Across all antibodies and dyads, plant-derived antibodies were present at significantly lower levels in neonates despite being present in dams at similar levels (Fig. 1B, inset).

### Plant-derived antibodies are well transferred across the placenta in mice.

**FIG 2:**
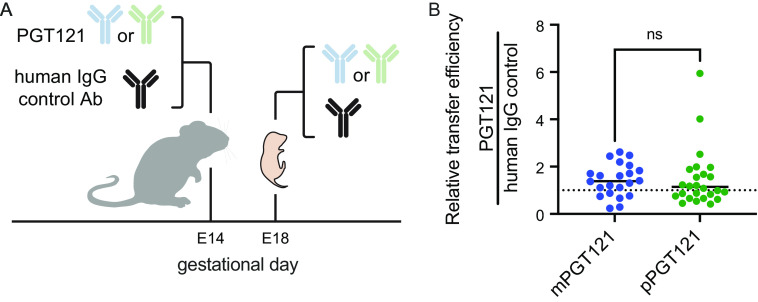
Plant-derived antibodies are well transferred across the placenta in mice. (A) Experimental schematic. Either plant- or mammal-derived PGT121 and a mammal-derived human IgG1 isotype control Ab were administered to dams (*n* = 2 to 3 per group) on embryonic day 14 (E14). Conceptuses were harvested at embryonic day 18 (E18), and Ab levels were quantitated in conceptus blood. (B) Relative levels of mammalian (blue) and plant (green) PGT121 compared to the coadministered human IgG control. The dotted line indicates equivalent transfer. Statistical significance was defined by Kruskal-Wallis test with Dunn’s correction. Bars indicate median values.

To evaluate whether plant-derived antibodies exhibited a general defect in placental transfer that would persist across species, their transplacental transfer properties were investigated in mice. Learning from the macaque experiments, to help account for biological variability between dams and pups and to additionally address the potential impact of differences in litter size, pregnant C57BL/6J mouse dams were coinjected with 50 μg of either mPGT121 or pPGT121 as well as 50 μg of a mammal-derived control human IgG (*n* = 3/group) at day E14 (Fig. 2A). An additional control group was treated with 50 μg of control IgG alone (*n* = 2). To avoid the confounding effect of nursing, animals were sacrificed 4 days post-Ab injection. Serum was harvested from the dams and their corresponding conceptuses, and Ab concentrations assessed by multiplex binding antibody assay. Statistically significant differences in the concentration of control IgG in conceptuses were not observed between mono- and coinfusion conditions, suggesting that this dose of antibody did not saturate the transfer process, nor were differences in control antibody transfer observed between plant and mammalian PGT121 coinfusion groups, suggesting that coinfusion of PGT121 did not influence the transfer of the control IgG. Differences in the median transfer between litters were generally smaller than differences between pups within each litter for the control antibody, indicating the value of this internal control. Relative to the pup-specific level of transfer of control IgG, plant- and mammal-derived PGT121 Abs were transferred similarly well (Fig. 2B). In sum, the roughly equivalent transfers of plant- and mammal-derived PGT121 Abs in mice suggest that the dramatic defect in placental transfer of plant-derived bNAbs observed in rhesus macaques does not generalize across mammals, but may instead be unique to primates or species with similar multilayer placental architectures.

### Distinct posttranslational modifications in plant-derived antibodies.

Several posttranslational modifications are known to impact antibody interactions with various receptors. First, oxidation of the methionine residue (Met252) present in the Fc region, previously observed in plant-derived antibody preparations (37), has been shown to have a negative impact on the IgG-FcRn interaction (38). To investigate whether methionine oxidation contributed to poor placental transfer, an initial analysis of all (residues 1 to 5) methionine sulfoxide residues on pPGT121-LS, pVRC07-523-LS, mPGT121, mVRC07-523-LS and m830A by nano-liquid chromatography–nano electrospray ionization–tandem mass spectrometry (nLC-NSI-MS/MS) was performed. Most methionine residues were in their native, nonoxidized form in both plant (92.3% to 100%) and mammalian (92.7% to 99.6%) Abs, eliminating variable methionine oxidation as an explanation for failure to transfer.

**FIG 3:**
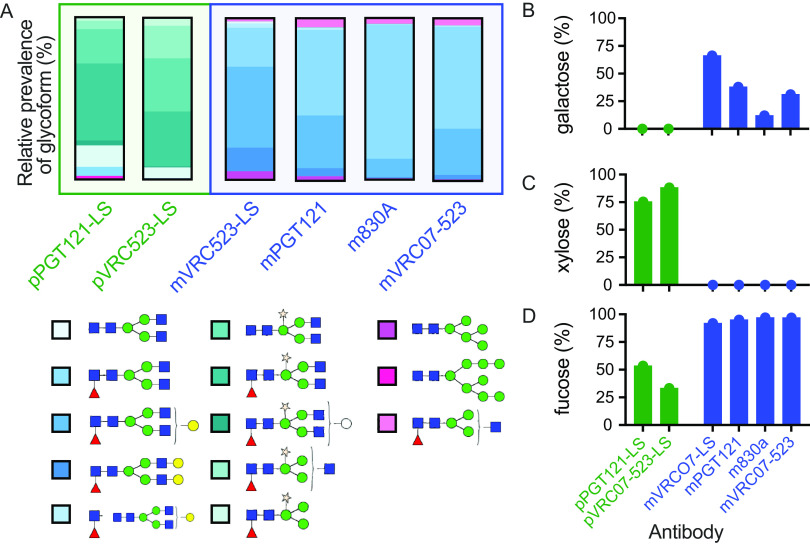
Distinct Fc glycan profiles of plant and mammal-derived antibodies. (A) Plant-derived PGT121-LS and VRC07-523-LS glycoforms were compared with mammalian PGT121, VRC07-523, and 830A glycoforms. All mammalian MAbs were produced in CHO cells except for 830A, which was produced in HEK293 cells. (B to D) Prevalence of different glycan features. (B) Glycan site nonoccupancy rate; (C and D) prevalence of xylose (C) and fucose (D). Note that plant-derived fucose has α,1-3 linkage and mammal-derived fucose has α,1-6 linkage. Red triangles, fucose (Fuc); blue squares, *N*-acetyl glucosamine (GlcNAc); green circles, mannose (Man); yellow circles, galactose (Gal); gold stars, β-1,2-xylose (Xyl); open circles, hexose (Hex).

Second, the presence and specific composition of an N-linked oligosaccharide at N297 on the Fc CH2 domain is critical for binding to FcγRs (39) and has been reported to modestly impact FcRn interactions (18, 40–43). Glycan analysis of the plant- and mammal-derived Abs used in the current studies was conducted (Fig. 3A) to determine whether or not each preparation exhibited the glycan profiles characteristic of its expression host. In plant-derived bNAbs, the Fc glycan sequon was ~75% occupied, and the predominant glycoform comprised a core structure of *N*-acetylglucosamine (GlcNAc) and mannose (12.7% and 6.4%, respectively), with the addition of β,1-2 xylose alone (20.2% and 31.5%) or β,1-2 xylose plus α,1-3 fucose (50.2% and 51.5%) for pPGT121-LS and pVRC07-523-LS, respectively. In contrast, mVRC07-523-LS, mPGT121, m830A, and mVRC07-523 Fc glycans consisted of the core structure appended with α,1-6 fucose and variably extended with mono- (G1) or digalactose (G2), as is typical of recombinant antibody production in mammalian cell lines.

Collectively, this analysis suggested that despite being known to impact FcRn binding, methionine oxidation was an unlikely driver of the poor placental transfer observed in macaques. In contrast, but as expected, plant-derived antibody glycans differed from mammalian glycans in lacking galactose (Fig. 3B), the type of complex glycans (xylose) appended during the late stages of N-glycosylation in the Golgi system (Fig. 3C), the prevalence and plant-specific α,1-3 linkage of fucose (Fig. 3D), the absence of galactose and sialic acid, and reduced glycan sequon occupancy. Combined with prior knowledge as to relative insensitivity of human FcRn to antibody glycosylation, but the high sensitivity of FcγR to Fc glycosylation, the extent of these differences suggested that distinct glycoprofiles might play a role in discrepant transfer efficiency in species in which FcγR contributes.

### Defect in placental IgG MAb transfer in primates is not associated with FcRn affinity.

**FIG 4:**
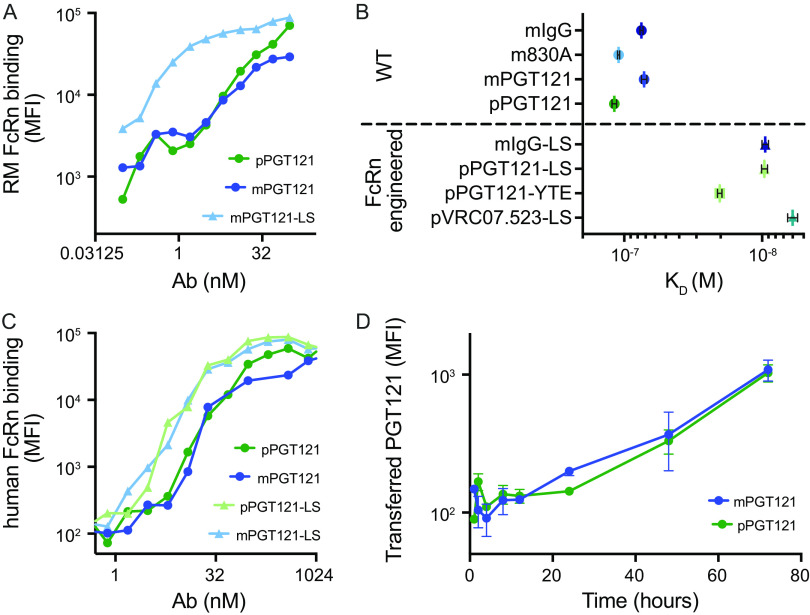
Defect in placental IgG MAb transfer in primates is not associated with FcRn affinity. (A and C) Binding of rhesus macaque (RM) (A) and human (C) FcRn to plant (p [green])- and mammal (m [blue])-derived antibodies in a microsphere assay; (B) kinetic binding affinity (*K_D_*) of mammal- and plant-derived Abs with and without FcRn affinity engineering mutations (LS or YTE) for RM FcRn tested by biolayer interferometry; (D) transfer of PGT121 across a monolayer of human placental epithelial (BeWo) cells over time. Error bars indicate standard deviation.

Because minor glycopreferences have been reported for primate FcRn, and because mouse and primate FcRn antibody binding preferences are known to be distinct (44), we next investigated whether poor transfer in primates was associated with compromised affinity for primate FcRn. Recombinant rhesus macaque FcRn was tetramerized and used to stain antibody-opsonized microspheres. FcRn affinity-engineered mPGT121-LS MAb showed elevated binding across a range of antibody concentrations, and plant- and mammal-derived PGT121 showed equivalent binding profiles (Fig. 4A). When the affinity between rhesus macaque FcRn and study antibodies was assessed by biolayer interferometry (BLI), both plant- and mammal-derived PGT121 and m830a were observed to exhibit a similar affinity to a control antibody (Fig. 4B), which was consistent with prior reports (45, 46). While the degree of affinity enhancement varied, FcRn affinity-engineered variants, whether expressed in plants (pPGT121-LS, pPGT121-YTE, and pVRC07-523-LS) or mammalian cells (mIgG-LS) exhibited higher affinity than Abs with unmodified Fc domains (Fig. 4B). A similar binding profile was observed for interactions with human FcRn. Affinity-engineered Abs showed elevated human FcRn binding signal, and plant- and mammal-derived antibodies showed equivalent binding in the microsphere assay (Fig. 3C). Thus, a defect in binding to primate FcRn failed to explain the poorer transfer of plant-derived antibodies in rhesus macaques.

To move beyond these binding assessments and capture a functional measure of placental transfer efficiency, we next assessed the ability of study antibodies to be transferred across a polarized human placental trophoblast monolayer, an *in vitro* system that models FcRn-dependent transfer across the syncytiotrophoblast (47, 48), which is the first of the three cellular layers separating maternal from fetal circulation. Plant-derived PGT121 was shown to cross this monolayer as well as its mammal-derived PGT121 counterpart (Fig. 4D), providing further evidence that there was not an FcRn-associated transfer defect in the plant-derived Abs. These results suggest that if FcRn were the sole gatekeeper of placental transfer in primates, efficient transfer would be expected of both plant and mammal-derived MAbs.

### Plant-derived bNAbs exhibit reduced binding to primate FcγRs.

**FIG 5:**
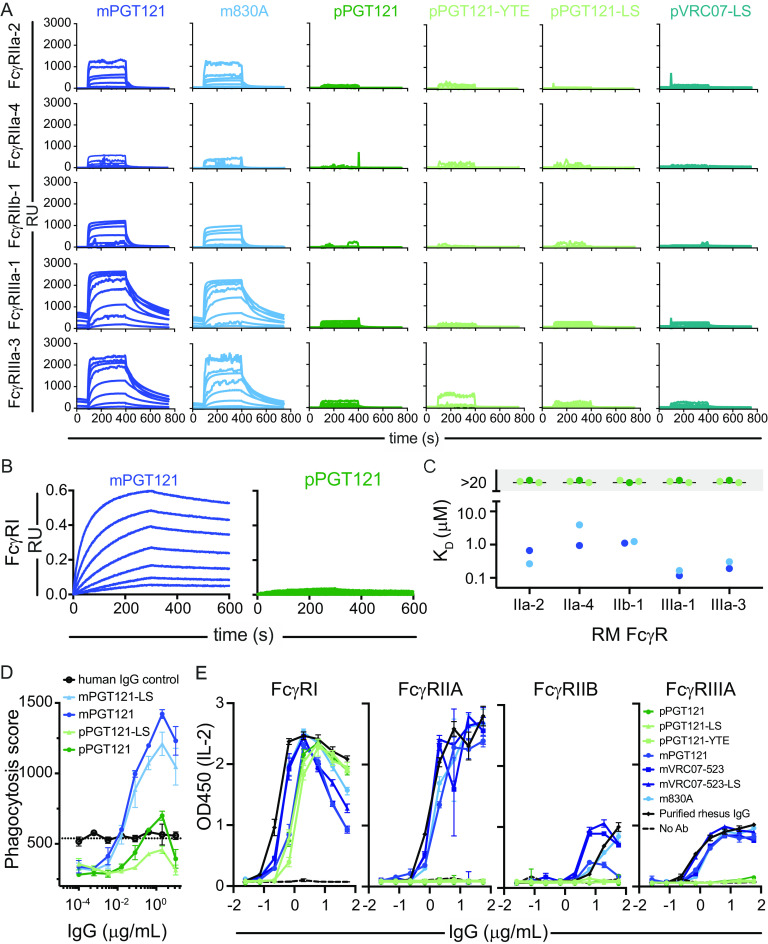
Poor placental IgG MAb transfer is associated with a reduced binding to primate FcγR. (A) Multiplexed SPR sensorgrams depicting association and dissociation of major low-affinity rhesus macaque FcγR allotypic variants (rows) across mammalian (m) and plant (p) antibodies (columns); (B) sensorgrams showing binding of the major high-affinity rhesus macaque FcγR1 variant to mammal- and plant-produced forms of PGT121 defined by BLI; (C) equilibrium binding affinities of antibodies across low-affinity FcγR. *K_D_* values that could not be confidently estimated were set as exceeding 20 μM, the highest concentration assessed. (D) Plant- and mammal-derived PGT121 and PGT121-LS were assessed for the ability to induce phagocytosis in human THP-1 cells. (E) Levels of IL-2 (optical density at 450 nm [OD_450_]) detected in supernatant of rhesus FcγR-expressing reporter cell lines induced by culture on antibody-coated plates.

While the expression of FcγR (FcγRI, FcγRIIIA, and FcγRIIB) in the human placenta is now well established, a role for receptors other than FcRn in placental transfer has remained controversial (9, 10, 17, 18, 25). Given their placental expression, we next evaluated whether a defect in the monovalent binding affinity to FcγR was associated with the poor placental transfer efficiency of plant-derived MAb observed in primates. Multiplex surface plasmon resonance (SPR) sensorgrams for mammal- and plant-derived antibodies showed a dramatically reduced ability of plant- but not mammal-derived antibodies to bind diverse low-affinity rhesus macaque FcγR (Fig. 5A). A similarly dramatic reduction in the ability of plant-derived PGT121 to bind the high-affinity macaque FcγR1 receptor was observed by BLI (Fig. 5B). Whereas both mammal-derived antibodies tested showed equilibrium binding affinities consistent with each other and prior literature (46, 49), the affinities of the plant-derived Abs were too low to be defined (Fig. 5C).

However, because even very-low-affinity interactions can drive effector functions in the context of high avidity, we next sought to further probe the apparent loss of FcγR binding of plant-derived Abs in the context of avid antibody interactions with FcγR in assays of phagocytosis and receptor-mediated signaling. First, plant- and mammal-derived PGT121 and the PGT121-LS mutants were compared for their ability to induce FcγRIIA-dependent phagocytic activity in the FcγRIIIA-negative THP-1 human monocytic cell line. Whereas mPGT121 and the mPGT121-LS mutant exhibited a robust ability to drive the uptake of fluorescent beads conjugated with recombinant HIV envelope glycoprotein, the phagocytic score of the plant Abs did not exceed that observed for negative controls, consistent with impaired FcγRIIA binding (Fig. 5D).

As a final means of assessing FcR interactions, we utilized a panel of BW5147 mouse T-cell lymphoma cells stably transfected to express a chimeric receptor containing a rhesus macaque FcγR (FcγRI, FcγRIIa, FγRIIb, or FcγRIII) ectodomain fused to the CD3ζ intracellular signaling domain (50). Engagement of this chimeric FcR with IgG leads to interleukin-2 (IL-2) secretion. With the exception of FcγRI, a high-affinity receptor, plant-derived Abs exhibited inferior binding and triggering of IL-2 production in these cell lines compared to the mammal-derived Abs and controls (Fig. 5E).

In sum, these data suggest that this deficiency in binding to FcγR is associated with the poor placental transfer phenotype observed for plant-derived antibodies. While plant- and mammal-produced antibodies exhibited comparable transfer efficiency in mice and consistent pharmacokinetic profiles in rhesus macaque dams, they were differentially transferred across macaque placentas. Coupled to similar binding to primate FcRn, these divergent placental transfer phenotypes point to the contribution of a second IgG transporter—potentially an FcγR—to transfer IgG across the primate placenta.

## DISCUSSION

Placentas are one of the most species-specific and rapidly evolving organs in the body (3), and this study suggests that their IgG transport mechanisms have diverged throughout mammalian evolution. The present study demonstrates that in contrast to mice, in which both plant- and mammal-derived HIV-specific IgG1 bNAbs exhibited comparable efficiencies of placental transfer, the macaque placenta was essentially unable (<0.1% efficacy) to transfer plant-derived bNAbs. Furthermore, LS and YTE variants, which exhibit increased binding affinity to primate and human FcRn and extended plasma half-life in both humans and macaques (51), only marginally increased transfer of plant-derived MAbs in macaques. While we presume that the relatively short period of exposure to recombinant antibody is a factor in the low transfer efficiency observed in rhesus macaques for all antibodies tested, the lack of placental transfer of plant-derived IgG could not be attributed to low Ab levels in treated dams. Similarly, while human and rhesus macaque IgG1s are highly homologous, and a number of distinct MAbs were employed, species-specific differences cannot be definitively excluded from playing a role in the low levels of even mammal-derived bNAb. The equivalence of the serum pharmacokinetic (PK) profiles between mammal- and plant-derived IgG1s, in contrast to the discrepant placental transfer phenotypes are surprising. In sum, this piece of data suggests that affinity for FcRn, despite being the only known IgG transporter, is insufficient to explain the inability of IgG1 to cross the macaque placenta. Data from this study clearly demonstrate that placental IgG transfer biology differs between species and indicates that the divergent evolution of the mouse yolk sac placenta to the three-layer placenta in humans may be associated with the exploitation by higher mammals of a second Fc receptor (52, 53).

While the identity of an additional placental Fc receptor that may contribute to transfer in higher mammals is currently not known, the presence of FcγRs on different human placental layers (19–23) provided the rationale to investigate these receptors. Though prior studies in mice have refuted a role of FcγR (10), centered on data in the primate model, we find that human IgG1 antibodies that fail to bind FcγR also fail to complete transfer to the fetal circulation in infant macaques. The simplest explanation for this observation is that FcγRs play a mechanistic role in antibody transfer across the placenta in primates.

Among candidate receptors, transfer patterns of IgG subclasses in humans are highly suggestive of a role for FcγR. Thus, while both human IgG1 and IgG2 bind FcRn well (54), the reduced transfer of IgG2 (55) is in line with its generally poorer binding to FcγR. Likewise, most IgG3 allotypes poorly bind to FcRn, but their presence in cord blood at levels the same as or greater than those in mothers (24) is in line with the high binding of IgG3 to FcγR. The expression of FcγRIIb on fetal endothelial cells (53) that comprise the final barrier to fetal circulation further validates a two-receptor model involving FcRn and FcγR, specifically FcγRIIb, rather than a model that relies on FcRn alone.

Whereas the primary amino acid sequences of paired plant- and mammal-derived PGT121 used in this work were essentially identical, their posttranslational modifications differed extensively. While differential oxidation was excluded as a potential driver of poor transfer, the lower Fc glycan site occupancy (75%), high level of xylose (70%), the presence of >50% of fucosylated Abs containing an α1-3 linkage unique to plant-derived antibodies, and the lack of galactose, thought by some to stabilize the IgG hinge region and potentially alter orientation of the Fc domains and reduce FcγR binding (56), were potentially associated with poor transfer phenotypes. In this context, in contrast to the exquisitely N297 glycan dependence of IgG-FcγR interactions, the pH-dependent interaction of FcRn with the CH2-CH3 interface of each Fc domain, appears to be for the most part, glycan independent (10, 25, 39). Thus, analysis in this and prior studies has demonstrated comparable binding of nonglycosylated and glycosylated unmodified and engineered glycoforms of plant-derived VRC01 (37) and other Abs (43) to human FcRn. While a subtle effect of galactose content on FcRn binding and/or placental transfer has been suggested from some (18, 40–43), but not other, studies (10, 25, 37, 57), our data demonstrate that variably glycosylated antibodies exhibit dramatically different transfer efficiencies, suggesting the involvement of a second glycan-dependent transporter.

In terms of maternal-fetal IgG transfer, higher frequencies of Fc fucose have been associated with poor placental transfer of anti-HIV gp120 Abs in HIV-infected mothers (8) and a slight shift in digalactosylated Abs has been associated with enhanced placental transfer in healthy women (18). In the current macaque study, glycan analysis of the mammalian Abs studied suggests that fucose and galactose levels did not appear to play dominant roles in MAb transfer efficacy. Mammalian antibodies had only moderate levels of mono- and digalactosylation, and m830A, which crossed the placenta most efficiently (18% transfer), had the lowest level of galactosylation. Similarly, despite containing >95% fucose, m830A and mPGT121 were efficiently transferred, consistent with the efficient transfer of fucosylated endogenous Abs observed in women immunized prior to or during pregnancy (58). This indifference to the extent of fucosylation observed in cord/infant versus maternal blood in human cohort studies (18) also favors a glyocopreference of FcγRII rather than FcγRIII.

Additional factors, including subtle changes to the kinetics of interactions with FcRn and FcγR competition between subclasses, may influence placental antibody transfer (59). For example, experimental systems in which FcRn function is assessed in the context of the cellular membrane have shown surprising impacts of the Fab domain (60). While a set of plant- and mammal-derived Abs with identical variable domains were evaluated here, a variety of studies have shown that conformational allosteric changes in Fc associated with properties of the Fv domain may impact both the quality of binding to both FcRn and FcγRs *in vitro* (56, 61–63). To this end, while the pPGT121 and pVRC07-523 Abs lack N-linked variable region glycans, 15 to 20% of IgGs in humans contain Fab glycans (64), which have been shown to be associated with variable transfer in pregnant mothers (65). Similarly, antibodies with different antigen specificities exhibit reproducibly distinct transfer efficiencies (8). Simple models equating FcRn affinity to transfer efficiency of FcRn affinity fail to fully explain these observations. Cumulatively, such studies clearly suggest that there are aspects of antibody transplacental transfer and recognition by diverse FcRs that are meaningfully impacted by factors beyond Fc domain sequence and glycosylation that remain incompletely understood. It should also be noted that even in humans, different MAbs can exhibit very different transfer levels from poor to high (0.04 to 20 μg/mL), although with persistent treatment, fetal levels appear to generally exceed those observed in mothers (66, 67).

Overall, this study points to differentiation in the basic mechanisms of inheritance of maternal antibodies among mammals and has important clinical implications. Monoclonal antibodies have become one of the most important and successful types of human therapeutics, and their use and impact during pregnancy have recently been extensively reviewed (67), revealing the different rates of transfer and clearance of different MAbs. Plant expression systems are fast and scalable and have become increasingly popular for the production of monoclonal antibodies, especially for time-critical applications. The failure of plant-derived Abs to efficiently cross the placenta in macaques provides a novel strategy for developing biosimilars of therapeutic Ab treatments that are contraindicated during pregnancy. Such therapies may be beneficial to the mother while preventing harm to the fetus and newborn.

## MATERIALS AND METHODS

### Transient plant expression of bNAbs.

Monoclonal antibodies (MAbs) PGT121 and VRC07-523 were produced by *Agrobacterium*-mediated transient gene expression in Nicotiana benthamiana (68). Synthetic codon-optimized variable domains were flanked by type IIs restriction sites and cloned into pTRAk plant expression vectors carrying the kappa constant domain as well as the LS-substituted (M428L N434S) IgG1 HC constant domain. The originally published PGT121 antibody amino acid sequences were used, but the VRC07-523 light chain contained an N90T mutation to prevent rapid clearance of the plant-derived form from the circulation (26). Briefly, 5- to 6-week-old plants were coinfiltrated with recombinant *Agrobacterium* suspensions individually carrying the pTRAk-based heavy- and light-chain expression plasmids and the pBIN-based p19 silencing suppressor from tomato bushy stunt virus. After 10 to 12 days, infiltrated leaves were harvested and soluble proteins were extracted and purified by protein A (Genscript, NJ) and MEP HyperCel mixed-mode chromatography (Pall Corporation, France), producing 600 to 1,200 mg/kg of leaf biomass, depending on the antibody. Purified bNAbs were stored at 4°C or frozen until use.

### NHP placental transfer experiments.

Six pregnant female Indian-origin rhesus macaques (Macaca mulatta) were assigned to the study from the Oregon National Primate Research Center (ONPRC) breeding colony and moved into animal biosafety level 2 (ABSL-2) housing within 4 weeks prior to birth. HIV bNAbs produced in mammalian or Nicotiana benthamiana plant cells were injected passively either singly or as cocktails by the subcutaneous (s.c.) route to the six dams close to parturition, as determined by sonogram, all receiving 5 mg/kg of each Ab. Since the precise time of parturition was not known, the length of time that the bNAb was in the dam was variable and some dams received a second injection to allow birth to proceed naturally (vaginally). Antibody concentrations in the dams and infants were determined at multiple time points by quantitative ELISA using standard curves for the matched antibodies. Four infants remained with their dams, while two infants separated from their dams on the day of birth were moved to the nursery and were bottle-fed formula.

The Oregon Health & Science University West Campus Institutional Animal Care and Use Committee approved all macaque studies. Studies were performed at the ONPRC in Beaverton, OR, USA, which complies with the Animal Welfare Act and Animal Welfare Regulations. The Center is accredited by AAALAC and adheres to the *Guide for the Care* and *Use of Laboratory Animals* (69) and U.S. regulations. Animals were monitored at least twice daily for behavior, food intake, activity, and overall health by trained animal care technicians. Newborns were not sedated for blood draws.

### ELISA.

Several types of ELISAs were performed to determine the pharmacokinetics of the administered bNAbs in the macaque dams and infants. Plates were coated with either RSC3 to measure VRC07-523 or ST0A9 to measure PGT121. Briefly, Costar high-binding plates (Corning) were coated overnight with 1 to 4 μg/well of antigen in carb/bicarb buffer (depending upon the antigen), washed with phosphate-buffered saline (PBS)–Triton X-100, and blocked with PBS with 5% milk and 1% normal goat serum for 1 h at room temperature (RT). Serial dilutions of all samples were compared with titrations of VRC07 or PGT121 as standard curves, and positive and negative controls were included on each plate. Plasma was incubated for 1 h at RT, followed by three washes with PBS–Triton X-100. Bound antibodies were probed with horseradish peroxidase (HRP)-labeled goat anti-human IgG (1:5,000 dilution) (Jackson Laboratories) for 1 h at RT. The plate was washed, and the TMB (3,3′,5,5′-tetramethylbenzidine) substrate (SouthernBiotech) was added. Once color was developed, stop buffer was added, and the optical density at 450 nm (OD_540_) was read. GraphPad Prism was used to calculate antibody concentrations and 50% binding titers (50% effective concentration [EC_50_]) of endogenous antibodies.

### Mouse placental transfer and pharmacokinetics experiments.

To evaluate placental transfer efficiency in mice, 50 μg of pPGT121 or mPGT121 was injected into C57BL6 dams (*n* = 3 per group) intraperitoneally on day 14 of parturition, along with 50 μg of mammalian cell-derived CH42 AAA (62), which is specific for glycoprotein D (gD) of herpes simplex virus (HSV), as an internal isotype control. Dams were sacrificed 4 days later, and serum samples were collected from the dams via cheek bleed and via decapitation for each conceptus. Procedures were performed in accordance with the policies of Dartmouth’s Center for Comparative Medicine and Research, following approval by the Institutional Animal Care and Use Committee. Antibody levels were quantified using a customized multiplex assay as described below. Due to the complexity of these studies, the full panel of MAbs was not evaluated.

### Multiplex Ab quantification.

Antibodies in serum were quantified by custom multiplex assay (70). Briefly, fluorescently coded magnetic microspheres (Luminex) were covalently conjugated with TRO gp120 (Immune Technology), cytomegalovirus (CMV) gB (Sino Biological), herpes simplex virus 1 (HSV-1) gD (Immune Technology), and goat anti-human IgM (Millipore Sigma). Mouse serum samples were diluted 1:100 in PBS and incubated with 1,000 of each type of microsphere (Luminex) in a 384-well plate (Grenier Bio One) at room temperature for 2 h with constant agitation. The assay plate was washed with assay wash buffer (PBS, 0.05% Tween 20, 0.1% bovine serum albumin [BSA]) using an automated plate washer (Biotek). The microspheres were then resuspended in assay wash buffer containing goat anti-human IgG Fc-phycoerythrin (PE) (SouthernBiotech) or goat anti-human IgM-PE (SouthernBiotech) at 0.65 μg/mL and incubated for 1 h. The assay plate was washed again, and then the samples were analyzed using a Luminex FlexMAP 3D (Luminex), and the median fluorescent intensity (MFI) for microsphere-bound PE signal was measured. Serum antibody concentrations were interpolated from a standard curve in Prism 9 (GraphPad).

### Methionine oxidation.

Methionine oxidation was analyzed on the glycoprotein samples of PGT121, 830A, VRC07-523, and VRC07-523-L by nano-liquid chromatography–nano electrospray ionization–tandem mass spectrometry (nLC-NSI-MS/MS). Twenty-five micrograms of each sample (1 mg/mL; 50 μL/vial) was used for glycoproteomic analysis. The samples were reduced via dithiothreitol (DTT) and alkylated via iodoacetamide before the proteins were enzymatically digested with trypsin at 37°C overnight. After inactivation of the trypsin, the solutions were filtered. The peptides and glycopeptides were analyzed as follows: samples were injected to an Orbitrap Fusion Tribrid mass spectrometer through a nano-LC system, and the glycopeptides were fragmented by a higher-energy collisional dissociation (HCD)-triggered collision-induced dissociation (CID) program (based on glycoform oxonium ions). The data sets were processed by Byonic software and further analyzed by manual annotation. For calculation of the relative percentages, the area under the curve of the most abundant peak (AUC^most abundant peak^) was calculated for each (glyco)peptide-derived mass. For each (glyco)form, the individual AUC^most abundant peak^ was divided by the sum of AUC^most abundant peak^ across all (glyco)forms.

### Glycan characterization.

Glycan analysis was performed at the Complex Carbohydrate Research Center (CCRC), University of Georgia. For glycoproteomic analysis, 25 μg of each MAb sample (1 mg/mL; 50 μL/vial) was reduced via DTT and alkylated via iodoacetamide before the proteins were enzymatically digested with trypsin at 37°C overnight, inactivated with trypsin, and filtered. The peptides and glycopeptide samples were analyzed as described above.

### FcRn binding microsphere assay.

An assay plate (Grenier Bio-one) was prepared with mammal- and plant-expressed versions of the following antibodies: PGT121, PGT121-LS, and VRCO7-LS, serially diluted 2-fold from 100 nM to 0.098 nM in assay wash buffer (PBS, 0.05% Tween 20, 0.1% BSA). The antibody samples were then incubated with microspheres (Luminex) conjugated with TRO gp120 (Immune Technology; IT-001-RB4p) for 2 h at room temperature with constant agitation. The samples were then washed using an automated plate washer (Biotek), resuspended in assay wash buffer at pH 6.2 containing streptavidin-PE (Agilent Technologies) tetramerized biotinylated rhesus FcRn at 0.65 μg/mL, and incubated for 1 h. The assay plate was washed again, and data were collected and analyzed as described above.

### FcRn binding biolayer interferometry assay.

Biolayer interferometry (BLI) on the Octet RED96 system (Sartorius AG) was used to characterize the 1:1 biophysical interaction between antibodies and rhesus FcγR (45), and both human and rhesus FcRn. For the measurement of the biophysical interaction with FcRn, protocols provided by the manufacturer of biotinylated recombinant rhesus FcRn (AcroBiosystems) were followed. Briefly, experiments were performed at 30°C in freshly prepared and filtered kinetics buffer (1× PBS, 0.1% BSA, 0.05% Tween 20) for rhesus FcγR1 or adjusted pH kinetics buffer (1× kinetics buffer [pH 6.0]) for FcRn association and dissociation. Biotinylated FcRn was immobilized using high-precision streptavidin-coated biosensors (SAX; Sartorius AG), and His-tagged FcγR was captured using biotin–anti-penta-His (Thermo) preimmobilized on next-generation streptavidin biosensors (SAX2; Sartorius AG) via a loading step with a 0.3-nm response unit (RU) threshold. Following a 60-s baseline step in kinetics buffer, loaded biosensors were dipped into 2-fold serially diluted antibody samples for a 60-s association step and subsequently for 60 s in kinetics buffer to measure dissociation. Antibody serial dilution ranges were as follows: 62.5 to 0.49 nM for FcγRI, 1,000 nM to 15.63 nM for low-affinity FcγRs and human FcRn, and 500 to 15.6 nM for rhesus FcRn. Immobilized FcRn was regenerated between antibody analytes by dipping 3 times for 5 s each into regeneration buffer (1× kinetics buffer [pH 7.4]), while immobilized FcγRs were regenerated between antibody analytes by dipping 3 times for 5 s each into regeneration buffer (10 mM glycine [pH 1.7]). Binding sensorgrams were aligned to the beginning of the association step for interstep correction and Y-aligned to the preassociation baseline step. Following a single reference subtraction consisting of immobilized receptor dipped into kinetics buffer, processed sensorgrams were globally fit to a 1:1 binding isotherm on ForteBio HT Analysis software (v.11.1.1.39) to determine kinetic constants.

### FcγR binding surface plasmon resonance assay.

Adapting a previously reported procedure, antibodies (mPGT121, pPGT121, pPGT121-YTE, pPGT121-LS, pVRC07-LS, and m830A) were covalently coupled to a carboxymethyldextran-functionalized sensor (CMD200M; Xantec Bioanalytics) using carbodiimide chemistry. A continuous flow microspotter (CFM) (Carterra) was used to print antibodies, with replicates, on a single sensor. The sensor surface was activated by a mixture of 10.4 mM EDC [1-ethyl-3-(3-dimethylaminopropyl) carbodiimide] (Thermo Fisher; 77149) and 2.8 mM sulfo-*N*-hydroxysuccinimide (Thermo Fisher) formulated in 10 mM MES (morpholineethanesulfonic acid [pH 5.0]), followed by the application of the antibodies at 100, 200, and 400 in 10 mM sodium acetate (pH 5.0). Ligand application ran for 10 min, followed by 5 min of washing with sodium acetate. The chip was then docked in the flow cell of an imaging-based surface plasmon resonance instrument (SPRi) (MX96; IBIS Technologies) for quenching with 1 M ethanolamine (Sigma-Aldrich). The remaining ligand was removed, and the overall capacity of the sensor was tested using successive injections (5 rounds in total) of 25 μg/mL anti-human Fab and 10 mM glycine (pH 3.0).

Rhesus macaque FcγR (IIa-2, IIa-4, IIb-1, IIIa-1, and IIIa-3) analytes were formulated in a running buffer consisting of 1× phosphate-buffered saline containing 0.05% Tween 20 at 20 μM and diluted 2-fold over an 8-point series. The receptors were injected from the lowest to highest concentration, and association with ligand was measured for 5 min, followed by 5 min of dissociation. Two blank injections of the running buffer following each receptor series were sufficient to completely dissociate these low-affinity analytes and prepare the sensor for another receptor.

Initial processing of the data was performed using SprintX (IBIS Technologies) and exported to Scrubber 2 (BioLogic Software). The signal from each region of interest on the sensor was referenced using the nearest unconjugated interspot to account for bulk shift and nonspecific binding. The blank injection immediately preceding each series of receptor was also subtracted from each concentration of receptor. Equilibrium affinity values for each receptor-antibody pair were calculated by regression with Prism 9 (GraphPad) using the average signal during a 10-s window at the end of the association phase for each analyte concentration when the system was at equilibrium. For cases in which the *R*_max_ could not be confidently defined from the dilution series, equilibrium dissociation constant (*K_D_*) values were plotted at >20 μM, the maximum concentration tested.

### Human placental cell Transwell transfer assay.

A previously reported Transwell transfer assay was used to define the ability of antibodies to cross a placental epithelial cell line monolayer (47, 48). Twenty-four-well cell culture plates with Transwell inserts (Thermo Fisher) were coated with human placental collagen (Sigma-Aldrich) by applying 50 μL of 1 mg/mL collagen solution to each well. The culture plate was allowed to dry in a biosafety cabinet and further sterilized by UV exposure for 30 min. BeWo cells (ATCC), a human epithelial placental cell line, were cultured in F-12 medium (Thermo Fisher) supplemented with 10% fetal bovine serum (FBS). A total of 1 × 10^5^ BeWo cells in 250 μL of medium (Thermo Fisher) were seeded in each well of the human placental collagen-coated Transwell plate and cultured overnight to establish a proper monolayer of cells, polarized by the placental collagen on the Transwell. Antibodies were added to the top chamber of the Transwell at a concentration of 40 nM. Human serum IgM (Millipore Sigma) and 1G2 (71), an IgG isotype control, were included in each well as internal negative and positive controls, respectively. To capture the longitudinal profile of transported antibody, 25-μL fractions were collected, with replacement, from the bottom chamber over time. Transferred antibodies in each fraction were quantified using the multiplex assay described above, with the additional inclusion of a goat anti-human IgM (SouthernBiotech; 9020-09) bead set. The MFI for each bead set was recorded and reported using GraphPad.

### Phagocytosis assay.

The phagocytic activity of antibodies was evaluated using a previously reported assay (72, 73). Briefly, fluorescent polystyrene 1.0-μm beads (Life Technologies) were conjugated with BaL HIV gp120 protein (Immune Technology) via cross-linking the carboxylic bead surface with primary amines on BaL HIV gp120. Assay plates were prepared with antibodies titrated from 10,000 ng/mL to 0.32 ng/mL in THP-1 medium (RPMI 1640 [Fisher] plus 10% FBS [Biowest]). BaL HIV gp120-coupled beads and THP-1 cells (ATCC) were added to the antibody-containing assay plate, at a 10:1 ratio of beads to cells for a total of 20,000 cells. The assay plate was incubated at 37°C under 5% CO_2_ for 4 h. Cells were washed in cold PBS, fixed with 4% paraformaldehyde, washed, and analyzed on a flow cytometer. Flow cytometry analysis included gating on cell populations in the forward scatter (FSC) versus side scatter (SSC) plot, followed by exporting the fluorescein isothiocyanate-positive (FITC^+^) signal MFI. A phagocytosis score was calculated by multiplying the MFI value by the percentage of FITC^+^. The human IgG1 used as a negative control does not bind to HIV gp120.

### FcR engagement via IL-2 reporter cells.

FcR engagement via IL-2 reporter cells was measured as previously described (50). In brief, high-binding 96-well plates (Greiner Bio-One) were coated with a 3-fold serial dilution starting at 50 μg/mL of each monoclonal antibody of interest in 0.1 M sodium bicarbonate (Sigma-Aldrich) buffer overnight at 4°C. Then, 50,000 BW5147 FcR-CD3ζ cells were added and cultured in RPMI (Gibco) supplemented with 10% (vol/vol) FBS (Sigma-Aldrich), sodium pyruvate (Sigma-Aldrich), and β-mercaptoethanol (Gibco) for 24 h at 37°C and 5% CO_2_. At the time that cells were added, 384-well high-binding plates (Corning 3700) were coated with 3 μg/mL unconjugated rat anti-mouse IL-2 (BD Biosciences) overnight at 4°C. Plates were washed once with 1× PBS (ScyTek) with 0.1% Tween 20 (Sigma-Aldrich) using an automatic plate washer (BioTek) and blocked for 1 to 2 h with 4% (mass/vol) whey (BiPro Elite), 15% (vol/vol) goat serum (Gibco), and 0.5% (vol/vol) Tween 20 in 1× PBS (Gibco) at RT. Following another single wash, supernatant from the BW5147 FcR-CD3ζ cultures was added directly to the IL-2 detection ELISA in duplicate and incubated at RT for 1 to 2 h. Mouse IL-2 (Roche) diluted in BW5147 culture medium was used as a positive control. Plates were washed once, biotin-conjugated rat anti-mouse IL-2 secondary Ab (BD Biosciences) was added, and the mixture was incubated for 1 h at RT. Following another single wash, streptavidin-HRP tertiary (Millipore) was added and the mixture was incubated for 30 min at RT. Plates were washed twice, and SureBlue (KPL, Inc.) was added for 3 to 5 min in the dark at RT. An equal volume of TMB stop solution (KPL, Inc.) was then added, plates were shaken for 5 s, and the optical density at 450 nm was read using a SpectraMax plate reader. Parental BW5147 cells without FcR-CD3ζ expression were used as a negative control to assess background IL-2 secretion.

### Data availability.

All primary biochemical and biological assay data in this article will be made available to investigators upon inquiry. Antibody sequences were published previously when antibodies were discovered.
